# How the Gut Microbiome Links to Menopause and Obesity, with Possible Implications for Endometrial Cancer Development

**DOI:** 10.3390/jcm10132916

**Published:** 2021-06-29

**Authors:** Malou P. H. Schreurs, Peggy J. de Vos van Steenwijk, Andrea Romano, Sabine Dieleman, Henrica M. J. Werner

**Affiliations:** 1 Department of Obstetrics, Gynecology and Gynecologic Oncology, Medisch Spectrum Twente, 7512 KZ Enschede, The Netherlands; 2 Maastricht University Medical Centre, Department of Obstetrics and Gynecology, GROW—School for Oncology and Development Biology, 6202 AZ Maastricht, The Netherlands; peggy.de.vosvansteenwijk@mumc.nl (P.J.d.V.v.S.); a.romano@maastrichtuniversity.nl (A.R.); erica.werner@mumc.nl (H.M.J.W.); 3 Maastricht University Medical Centre, Department of Surgery, GROW—School for Oncology and Developmental Biology, 6202 AZ Maastricht, The Netherlands; s.dieleman@student.maastrichtuniversity.nl

**Keywords:** endometrial cancer, gut microbiome, menopause, obesity, estrogen

## Abstract

**Background**: Interest is growing in the dynamic role of gut microbiome disturbances in human health and disease. No direct evidence is yet available to link gut microbiome dysbiosis to endometrial cancer. This review aims to understand any association between microbiome dysbiosis and important risk factors of endometrial cancer, high estrogen levels, postmenopause and obesity. **Methods**: A systematic search was performed with PubMed as primary database. Three separate searches were performed to identify all relevant studies. **Results**: Fifteen studies were identified as highly relevant and included in the review. Eight articles focused on the relationship with obesity and eight studies focused on the menopausal change or estrogen levels. Due to the heterogeneity in patient populations and outcome measures, no meta-analysis could be performed. Both the menopausal change and obesity were noted to enhance dysbiosis by reducing microbiome diversity and increasing the Firmicutes to Bacteroidetes ratio. Both also incurred estrobolome changes, leading to increased systemic estrogen levels, especially after menopause. Furthermore, microbiome dysbiosis was reported to be related to systemic inflammation through toll-like receptor signaling deficiencies and overexpression of pro-inflammatory cytokines. **Conclusions**: This review highlights that the female gut microbiome is intrinsically linked to estrogen levels, menopausal state and systemic inflammation, which indicates gut microbiome dysbiosis as a potential hallmark for risk stratification for endometrial cancer. Studies are needed to further define the role the gut microbiome plays in women at risk for endometrial cancer.

## 1. Introduction

Excess exposure to menopause, obesity and excess exposure to estrogen have long been recognized as risk factors important in endometrial cancer development; however, the exact mechanism and the molecular interplay of these components remain to be elucidated [1,2,3]. In addition, not all women with excess exposure to estrogen or obesity will suffer from endometrial cancer. This suggests that these factors induce varying effects on the endometrium or that they most likely do not explain the complete phenotype. A possible explanation for the observed discrepancy between known risk factors of endometrial cancer and varying outcomes may be the interference of the human microbiome [4,5]. Research on risk stratification for endometrial cancer development is important because incidence rates of endometrial cancer are still rising steadily and are predicted to continue to increase in the upcoming years [1]. 

The human microbiome is the largest organ of the human body, one of the most complex ecosystems colonized by more than 100 trillion micro-organisms [6]. It resides on and within human tissues throughout the body and in health in equilibrium with the human cells at the corresponding anatomical sites [7,8]. Although stable over long periods, the composition and functions of the human microbiome may be influenced by a number of factors including genetics, mode of delivery, age, diet, BMI, geographic location, and medical treatments including antibiotics [6,7,8]. The microbiome comprises many organisms with oncogenic, hormonal, metabolic and inflammatory potential, thus playing an important role in health and disease [9,10,11,12]. With the development of advanced methods of sequencing technologies using RNA and DNA directly extracted from fecal samples, such as 16S ribosomal RNA gene sequencing, we are able to gain a better understanding of the role and effects of the gut microbiome both in symbiosis and dysbiosis [13].

In volume, the gut microbiome is the largest bodily microbiome and accounts for more than 90% of the total human microbiome [14,15,16]. It consists of more than 500 different types of bacteria, creating a complicated and fragile ecosystem [14,15]. The main phyla of bacteria in the gut microbiome are Firmicutes, Bacteroidetes, Proteobacteria, Actinobacteria, and Verrucomicrobia of which the Firmicutes and Bacteroidetes make up 90% [17,18,19,20]. High diversity defines a healthy human gut microbiome, whereas reduction in diversity may be associated with dysbiosis. Dysbiosis refers to an altered microbiome composition that results from an abnormal balance between commensal and pathogenic bacterial species. Many studies have suggested a possible direct relationship between dysbiosis and inflammatory and metabolic diseases, obesity and cancer [7,8,14].

Gut dysbiosis caused by a higher Firmicutes to Bacteroidetes ratio has been associated with an increased risk for developing obesity [21]. This dysbiosis between the Firmicutes and Bacteroidetes phyla has also been shown to lead to an increase of bacteria with beta-glucuronidase (ß-glucuronidase) activity, which is an enzyme residing in microbes influencing estrogen metabolism. All microbes with this capability are collectively referred to as the estrobolome [22,23,24,25]. Beta-glucuronidase is able to deconjugate estrogen metabolites, leading to their reabsorption into the circulation, resulting in elevated levels of circulating estrogens [22,23,24,25,26].

So far, no studies correlating gut microbiome to endometrial cancer have been published. However, recent studies have suggested that local uterine microbiome dysbiosis may increase the risk of endometrial hyperplasia and cancer, in which different phyla play an essential role [27,28,29]. However, in these studies, the much larger gut microbiome was not investigated; thus, only local effects were considered to influence this risk. More importantly, the immense gut microbiome may, due to its intricate relationship with systemic steroid hormone levels, affect the risk of multiple estrogen induced diseases such as endometrial cancer or estrogen dependent breast cancer [23,25].

In this review, we searched the current literature that investigated and possibly linked estrogen levels, menopausal status and/or obesity, to gut microbiome composition and function. By further unraveling the intricate relationship between these known risk factors for endometrial cancer and gut dysbiosis, we may be able to outline future research into the correlation between gut microbiome and endometrial cancer and identify possible targets in prevention and therapy for this disease.

## 2. Data Selection and Extraction

### 2.1. Literature Search

A systematic review was carried out using PubMed as primary database to identify relevant literature. All searches were performed between May and July 2020 to identify articles studying the relationship between gut microbiota (dysbiosis) and endometrial cancer risk factors: estrogen, postmenopausal status and obesity. We performed 3 separate searches using the following combination of key words:

1. “Estrogen”, “Estradiol”, “Sex steroid hormones” and varieties of “Gut microbiome”.
2. “Menopause”, “Postmenopause, “Postmenopausal women”, “Postmenopausal” and varieties of “Gut microbiome”.
3. “Obesity”, “Obese”, “obese women” “overweight”, “Overweight women”, and varieties of “gut microbiome”.

In addition, we confirmed the lack of direct evidence on gut microbiome and endometrial cancer using the following key word combination “Endometrial Neoplasms”, “Uterine Neoplasms”, “Endometrial cancer” “Uterine cancer”, “endometrial hyperplasia” and varieties of “Gut microbiome” (search 4 in Supplemental File).

The reference lists of all selected studies were manually searched. No publication period restrictions were imposed; however, we limited our search to English literature only. Full details of the search strategy is provided in Supplemental File S1.

### 2.2. Eligibility Criteria

The direct search on endometrial cancer and gut microbiome confirmed that there is indeed no direct evidence available. For the other searches, we included all articles investigating the influence of estrogen, menopausal status and obesity on the gut microbiome. Both human and animal studies were included. Articles researching gut microbiota in research participants already receiving cancer treatment were excluded. We selected studies that included a control group to have comparative data, so that we can analyze the changes in microbiome due to estrogen/menopausal status or female obesity. Furthermore, we excluded literature that studied the possible influence of different diets on the gut microbiome and studies investigating other malignancies or gynecologic pathologies related to gut microbiome. Finally, we excluded studies when their results were not stratified for gender.

### 2.3. Data Extraction

The studies were selected by two individual authors (M.P.H.S. and H.M.J.W.) by reviewing titles, abstracts and keywords for relevance to the different risk factors for developing endometrial cancer and the gut microbiome. After this first selection process, the full text of the selected articles was obtained to assess their definitive eligibility. Reference lists were scrutinized for further relevant literature. Any disagreements were resolved through discussion between these two authors. When available, estrogen levels, menopausal status, BMI, alpha diversity and Firmicutes to Bacteroidetes ratio were extracted. Furthermore, first author, publication year, study design and sample size were extracted (Table 1 and Table 2).

### 2.4. Quality Assessment and Data Synthesis

We assessed the methodological quality of the included studies on the basis of the Newcastle-Ottawa Scale (NOS) methodology for the case control and cohort studies [30] (Supplemental File S2). To assess risk of bias for cross-sectional studies, the Appraisal tool for Cross-Sectional Studies (AXIS) methodology was applied, and last, for animal studies, the Systemic review center for laboratory animal experimentation (SYRCLE’s) tool for bias was used [31,32] (Supplemental File S2). For alpha diversity, we used the data that were calculated using Shannon-index or Operational Taxonomic Units (OTU) [33]. The Shannon index is a pure diversity index. It measures how evenly the microbes are distributed in a sample. OTU is the amount of taxonomic units present and thus measures differences in units, e.g., diversity. A statistician was consulted for evaluating statistical heterogeneity and questioning possible statistical analysis.

## 3. Outcome

### 3.1. Estrogen, Menopausal Status and Gut Microbiome 

#### 3.1.1. Literature Search

We combined the results for the first and second search because the latter only generated duplicates from the second search. Thus, the second and third search combined provide a complete overview of studies focusing on the correlation between menopausal status and estrogen levels and gut microbiome. A total of 188 studies of possible interest were considered out of 286 hits screened on title, abstract and keywords. In total, 154 articles were discarded as they either did not discuss the topic of interest or did not have an appropriate control group. The articles that remained included after the first selection, 34 full text articles were further evaluated. A total of 26 articles were discarded based on the fact that these articles discussed microbiome in general, dietary interventions, or did not include healthy individuals. Eventually, 8 articles were included, presented in Table 1. Among these studies, 5 involved human patients, and 2 involved animals (rodents).

#### 3.1.2. Quality and Risk of Bias of Selected Studies

Using NOS and AXIS, the selected articles demonstrated fairly to good quality (Table 3). The animal studies were graded using SYRCLE’s risk of bias tool.

#### 3.1.3. Main Outcomes

##### Alpha Diversity

From the 8 included studies, 6 reported on the alfa diversity of gut microbiota from premenopausal women compared to postmenopausal women [34,35,36,37,38]. In all 6 studies, the Shannon index was used to calculate the alpha diversity. Three studies [35,37,39] demonstrated that alpha diversity was significantly lower in the postmenopause compared to the premenopausal state (*p* < 0.05), while 2 other studies did not find any differences in alpha diversity when postmenopausal patients were compared to the premenopausal patients [36,38].

##### Firmicutes to Bacteroidetes Ratio

Firmicutes and Bacteroidetes are considered the most abundant phyla of the gut microbiome. Thus, differences in abundance of these phyla may seriously affect symbiosis and have consequences for gut microbiome functioning and, thereby, possibly play a role in the causation of certain related health conditions. Five studies reported on the presence of Firmicutes and Bacteroidetes [35,36,37,39,40]. All noted a disturbance in this ratio comparing pre- and postmenopausal women or women with high and low estrogen levels, suggesting that this ratio may be contributing to maintaining homeostasis in estrogen metabolism, possibly providing a therapeutic target in estrogen-related diseases. Four studies found that in postmenopause, the relative abundance of Firmicutes increased, and the presence of Bacteroidetes decreased, resulting in a significantly higher Firmicutes to Bacteroidetes ratio. This suggests that menopausal changes may induce gut dysbiosis, leading to a higher risk to develop health-related problems [35,38,39,40]. On the contrary, a recent study from Zhao et al. demonstrated that the Firmicutes to Bacteroidetes ratio decreased due to both a decrease in Firmicutes and an increase in Bacteroidetes [37]. Interestingly, the women included in this study were found to have the lowest BMI among all the study groups, which may have contributed to these conflicting results.

##### Family and Genus

**Figure 1:**
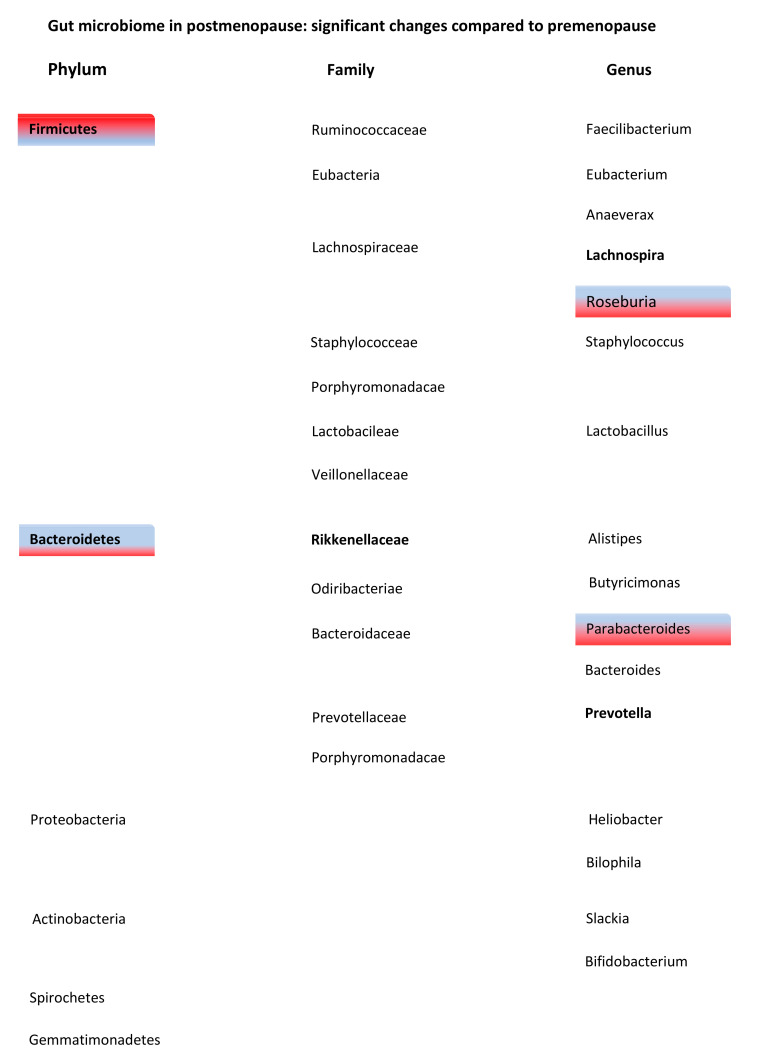
Changes in microbes of the gut microbiome from postmenopausal women compared to premenopausal women. Phyla are divided in families and genera. Red corresponds with an increase and blue corresponds with a decrease in relative abundance of phylum, family or genus in postmenopausal women. No color represents that this phylum or family was not specifically mentioned in the literature. When text font is bold, more than one article found this effect.

Phyla have several orders in their taxonomic rank that include many families, which again consist of different kinds of genera. All studies, except for one [37], found a higher abundance of most families and genera that belonged to the phyla Firmicutes in the postmenopausal group, also in line with the shift in the Firmicutes to Bacteroidetes ratio. Moreover, those same studies showed less abundance of the families/genera in the phyla Bacteroidetes in the postmenopausal group. All changes in phyla, families and genera in postmenopause are visualized in Figure 1.

In the phylum Firmicutes, Shin et al. demonstrated that in premenopause, the predominant families were *Ruminococcaceae* (42.3%), *Lachnospiraceae* (39.9%) and *Veillonellaceae* (11.7%), of which *Veillonellaceae* significantly decreased and *Lachnospiraceae* significantly increased after menopause [35]. Furthermore, an important genus, *Roseburia*, thought to be important in metabolic and endocrine disease, is reported to have either a higher or lower abundance in postmenopause [37,39]. Interestingly, these 2 studies differ in BMI. The study with only lean women demonstrated a decrease in *Roseburia* in menopause and the study that only included women with a BMI higher than 25 showed an increase in *Roseburia* after menopause [37,39]. Interestingly, 2 other studies reported that *Lachnospiraceae*, a family that has local anti-inflammatory effects through induction of regulatory T cells, is more abundant after menopause compared to the premenopausal state (*p* = 0.047) [35,39,41]. The last main observation in the human studies included that the presence of the genus eubacterium, which is located with the isoflavones, after menopause possibly prevents the onset of dysbiosis [37]. Regarding the included animal studies, ovariectomized rats demonstrated an increased abundance of *Lactobacillus* species (*p* = 0.049) and the exclusive presence of *Bifidobacterium animalis* (*p* = 0.043) compared to premenopausal rats [38,40].

Within the Bacteroidetes phylum, one study showed a dominant presence in the gut microbiome of the families *Bacteroidaceae* (61.2%), *Prevotellaceae* (28.6%) and *Rikenellaceae* (3.6%) [35]. The same study also demonstrated that the relative abundance of *Bacteroidaceae* was higher in the premenopause, although in absolute numbers the *Rikenellaceae, Porphyromonadaceae* and *Odoribacteraceae* families were more abundant in postmenopausal women compared to premenopausal women. The relative abundance of the genera *Parabacteroides* (*p* = 0.002), *Prevotella* (*p* < 0.001) and *Bilophila* (*p* not stated) were lower in postmenopausal women than in premenopausal women [39]. In contrast, Zhao et al. found that the family *Bacteroides* had a higher abundance in postmenopausal compared to the premenopausal women in line with their reported ratio in Firmicutes to Bacteroidetes ratio [37]. Moreover, the relatively new genus *Alistipes* was found to be lower in postmenopause. The animal studies reported that ovariectomized rats had a significantly lower abundance of the genera prevotella and Bacteroides compared to the non-ovariectomized rats [35,36,40].

##### Estrogen-Gut Axis

Next, we looked into a possible connection between estrogen metabolism and the gut microbiome. Most studies (*n* = 6) reported about this relationship [35,37,38,39,40]. Postmenopausal women seem to have increased deconjugation of estrogens demonstrated by a significantly higher level of parent estrogens compared to estrogen metabolites (2-, 4- and 16-hydroxilated metabolites) in the urine of postmenopausal women compared to the urine of premenopausal women [34]. Fuhrman et al. also found that non-ovarian estrogens, for example, estrogens produced by cells of adipocyte tissue, were strongly inversely associated with fecal microbiome richness and alpha diversity [34]. This correlation between the levels of estrone and microbiome diversity may imply that more estrogens are excreted through feces when microbial diversity and enzymatic activity are low. Flores et al. demonstrated that in premenopause, estrogens were not associated with taxonomic relative abundance at the phylum level however strongly associated with each other in postmenopausal women [42]. Non-ovarian urine estrogen levels were strongly and significantly associated with *Clostridia*, the most abundant family in Firmuctes, non-*Clostridiales* and three genera in the family *Ruminococcaceae* (β = 0.57 to 0.70, *p* = 0.03 to 0.002) [42]. These associations were primarily driven by levels of the parent estrogen estrone and estrone metabolites, with estrone being the most abundant estrogen after menopause. No correlation was reported between total premenopausal estrogens and abundance of non-*Clostridiales* of the phylum Firmicutes [42].

**Figure 2:**
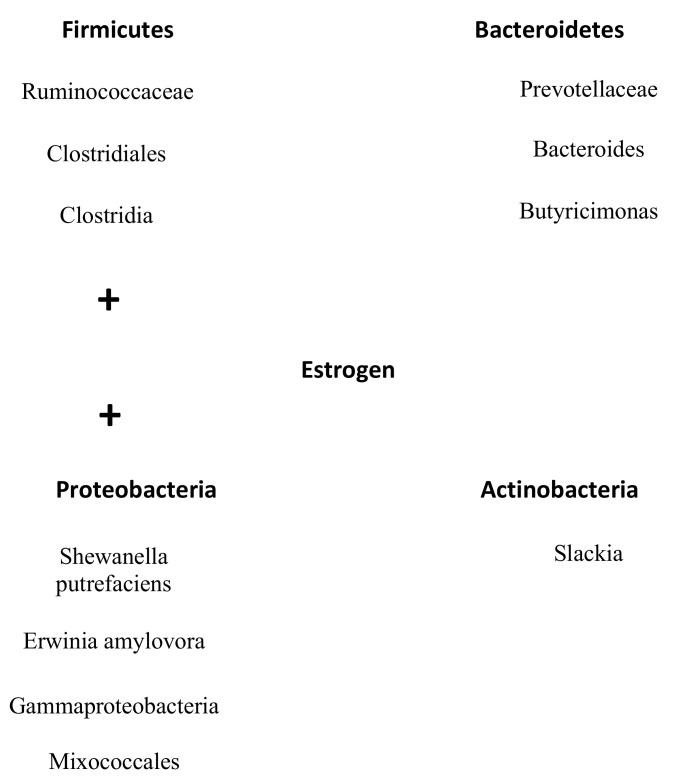
Overview microbes of the gut microbiome that correlates with estrogen levels. All families and genera from the phyla Firmicutes and Protobacteria positively correlate with estrogen levels. On the contrary, all documented families and genera from the phyla Bacteroidetes and Antinobacteria are negatively correlated with estrogen levels.

In addition, Santos-Marcos et al. demonstrated that some bacterial taxa outside of the phyla Firmicutes and Bacteroidetes, namely the *Gammaproteobacteria* class and a not-classified family from *Mixococcales*, positively correlated with estradiol levels (R = 0.575, *p* = 0.013 and R = 0.521, *p* = 0.039, respectively) [39]. Moreover, the bacterial family *Prevotellaceae* negatively correlated (R = −0.523, *p* = 0.018) with estradiol levels [39]. Choi et al. further showed that *Slackia* (R = −0.4, *p* = 0.033) and *Butyricimonas* (r = −0.4, *p* = 0.046) were negatively correlated with serum estradiol levels [40]. All families and genera that influenced the levels of estrogens are visualized in Figure 2. Overall, it becomes clear that bacteria that belonged to the phylum Firmicutes increasing levels of estrogen and that bacteria from Bacteroidetes have a reversed effect. Furthermore, the smaller phylum Proteobacteria had the same effect as Firmicutes and Actinobacteria had the same influence on estrogen as the phylum Bateroidetes (Figure 2/Table 1).

The ratio of estrogen metabolites to parent estrogen was correlated with order (taxonomic rank above family) *Clostridiales* (R = 0.32, *p* = 0.02) and family *Ruminococcaceae* (R = 0.37, *p* = 0.05). The genus *Bacteroides* was inversely associated with the ratio of estrogen metabolites to parent estrogens (R = −0.3, *p* = 0.03). Associations were independent of age and BMI [34].

Furthermore, when looking at the relationship between urinary and total fecal estrogen levels, a negative correlation was found (R = −0.43, *p* = 0.02) [42]. This inverse association with urine estrogens was especially strong for deconjugated fecal estrone (R = −0.50, *p* = 0.005) [42]. Conjugated estrogens and metabolites in feces were not significantly correlated with urinary estrogen levels [42]. Moreover, one study examined the activity and possible relationship between the enzyme ß -glucuronidase and levels of estrogens in urine [42]. This enzyme is responsible for deconjugation of estrogens in the gut, resulting into in their reabsorption and thus a larger amount of systemic deconjugated estrogens [43]. It was found that ß-glucuronidase activity was inversely correlated with both deconjugated and conjugated estrogens in feces (*p* ≤ 0.01). Additionally, fecal ß-glucuronidase activity was significantly correlated with urinary estrone levels (R = 0.36, *p* = 0.04) but not with total urinary estrogens (R = 0.24, *p* = 0.19), estradiol (R = 0.16, *p* = 0.38) or estrogen metabolites. Activity of the control enzyme, ß-glucosidase, was not related to total urine estrogens (R = 0.12) or to any of the parent estrogens or metabolites. In premenopausal women, urinary estrogens were not correlated with either ß-glucuronidase or ß-glucosidase activity, suggesting that ß-glucuronidase is predominantly important in gut dysbiosis in postmenopausal women [42].

Taking into account the growing evidence that oncogenesis in many cancers including endometrial cancers may be characterized by a pro-inflammatory state, levels of inflammatory cytokines related to the hormonal status in women are discussed although only reported in one study [39]. In this study, one observed higher interleukin-6 (IL-6) and monocyte chemoattractant protein- 1 (MCP-1) plasma levels in healthy postmenopausal women compared to the premenopausal state (*p* = 0.036 and *p* = 0.045, respectively) [39]. Moreover, tumor necrosis factor-alpha (TNF-alpha) increased in postmenopause, although this was not statistically significant.

### 3.2. Obesity in Women and Gut Microbiome

#### 3.2.1. Literature Search

For the third search, 602 articles were initially found. Most of these articles described either obesity in correlation with microbiota in conjunction with the risk of colon carcinoma, the effect of the gut microbiota after gastric bypass, or gut microbiota changes after introduction of specific food elements. These articles were excluded. Articles that did not differentiate between men and women were also excluded, since the aim of the search was to find differences in obesity and gut microbiota that could be relevant in the development of endometrial cancer, and sex-specific differences were thus deemed important. After screening all articles, the full texts of 56 articles were evaluated. Ultimately, only 8 articles were included that described the direct relationship between the gut microbiota and obesity in women only. These studies are described in Table 2. Table 2a includes 6 studies with women only and Table 2b describes 2 studies with sex-specific analyses.

#### 3.2.2. Quality and Risk of Bias of Selected Studies

Using the NOS the selected articles demonstrated fairly to high quality (Table 3). The included animal study was graded using SYRCLE’s risk of bias tool.

#### 3.2.3. Main Outcomes

##### Alpha Diversity

In most studies, the Shannon index or operational taxonomic unit (OTU) was similar between the groups of women with high BMI and women with low BMI. Menni et al. investigated effects on gut microbiome related to spontaneous differences in weight over a period of years and found a negative correlation between alfa diversity and increase in BMI [44]. Choi et al. found a lower alpha diversity in mice that were fed a diet high in fat, which was more pronounced in ovariectomized compared to non-ovariectomized mice [40]. When investigating fat distribution patterns, Min et al. (Table 2b) revealed a negative association between gynoid fat ratio (the amount of gynoid fat in relation to total fat distribution) and alpha diversity in all women [45].

##### Firmicutes to Bacteroidetes Ratio

Four studies reported about the specific phyla Firmicutes and Bacteroidetes and changes in their ratio to one another relative to differences in BMI [40,46,47,48]. They found that abundance of the phylum Firmicutes was increased in obese women and that abundance of the phylum Bacteroidetes was somewhat decreased in obesity, ultimately resulting in an increased Firmicutes to Bacteroidetes ratio in obese women [40,46,47,48]. The presence of metabolic syndrome showed a similar increase in this ratio [46]. Moreover, a large neck circumference, measured in this study as a proxy of fat distribution, led to an increase in Firmicutes, suggesting fat distribution along increased BMI is also relevant [47]. These observations again indicate that obesity is multifactorial and that also gut microbiome dysbiosis is linked to obesity. Moreover, Haro et al. demonstrated that the Firmicutes to Bacteroidetes ratio increased more in obese women compared to obese men, suggesting this link is sex-specific [49].

##### Family and Genus

**Figure 3:**
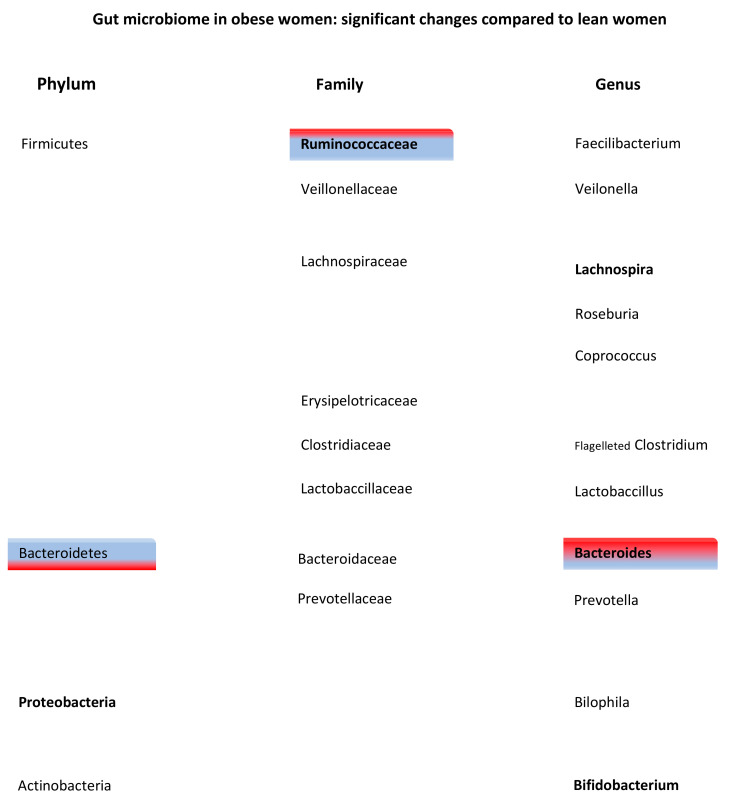
Changes in microbes of the gut microbiome in obese women compared to lean women. Phyla are divided in families and genera. Red corresponds with an increase and blue with a decrease in relative abundance of the phylum, family or genus in obese women. No color represents that this phylum or family was not specifically mentioned in the literature. When text font is bold, more that one article has found this effect.

Overall, families and genera that belong to the phylum Firmicutes were more abundant in obesity, suggesting that specific shifts in bacteria occur in obesity (Figure 3). The genus *Faecalibacterium* from the phylum Firmicutes was found to be more frequently present in obese women and even more often in obese women with metabolic syndrome (*p* = 0.0003) [46]. In addition, 5 different OTUs from the phylum Firmicutes decreased in abundance when women lost weight. Three of these OTUs belonged to the family *Lachnospiraceae* [50]. Lastly, the genus *Roseburia*, which has been thought to be a biomarker in several metabolic diseases [51], is significantly more abundant in obese women. In contrast, only the family *Ruminococcaceae*, from the phylum Firmicutes, was found to be nominally protective against weight gain and obesity (OR 0.89, *p* = 0.38) [44,50]. Two studies containing premenopausal women and ovariectomized rats demonstrated a significant decrease in the genus *Bacteroides* in obesity [40,46], whereas one study that included perimenopausal women found an increased abundance of this genus [44] (Figure 3).

When comparing men and women, in order to highlight the specific interactions in the gut microbiota of women, differences in relative abundance of several genera were present [45,49]. In obese women, the genera *Bilophila* and *Veillonella* were more present in the gut microbiota compared to obese men (from the phyla *Proteobacteria* and *Firmicutes*, respectively) [49]. In men, genus *Methanobrevibacter*, from the smaller phylum Euryacheota, was more abundant and may play a role in gut dysbiosis specifically in obese men, suggesting other interactive dynamics in women compared to men.

When looking at the influence of fat distribution, gynoid fat (fat around hips, thighs and breasts), but not android fat, seems to be an important factor in gut dysbiosis [45]. Since women and men have different fat distribution, this supports the gender differences. Min et al. demonstrated a positive association between the family *Ruminococcaceae* and presence of gynoid fat in women, but a negative effect in men. Moreover, the family *Provotellaceae* from the phylum Bacteroidetes showed the largest positive association with the gynoid fat levels. Again, this effect was only present in women [45].

Lastly, Pekkala et al. found that obese women had a significantly higher toll like receptor 5 (TLR5) gene expression and more often had gut microbiota dysbiosis compared to their counterparts. Toll like receptor 5 is a protein encoded in TLR5 gene, present on flagellin bacteria in the gut microbiome. These findings suggest that TLR5 signaling may be a link between gut dysbiosis and obesity in women [48]. Miranda et al. presented data on the correlation between higher waist/hip circumference ratio and was related to a higher presence of the phylum firmicutes and obesity and inflammatory markers. Women with a high weight circumference (e.g., intra-abdominal fat and thus apple shape fat distribution) had increased systemic inflammatory markers, such as highly sensitive c-reactive protein (hsCRP), TNF-alpha and leptin. The effect size of leptin was high and from hsCRP and TNF-alpha moderate. However, no direct link between these markers and gut microbiome was presented.

## 4. Discussion

This review focuses on the link between the gut microbiome composition and function, and the important risk factors for the development of endometrial cancer (menopausal/estrogen status and obesity in women). To date, there is no literature that investigates the direct influence of gut microbiome on endometrial cancer; however, this review shows that menopausal status and female obesity are correlated to gut microbiome dysbiosis, identifying possible targets for future research and a starting point for studies directly exploring the relation between endometrial cancer and the gut microbiome.

Estrogen acts on the tissues in the lower female reproductive tract, amongst others increasing intra-uterine epithelial thickness, glycogen levels and mucus secretion [26]. Dysbiosis in the gut microbiome, characterized by low gut microbiome diversity, potentially contributes to the disruption of this homeostasis by altering the estrobolome, thereby altering systemic estrogen availability, usually enhancing it [52,53]. Microbially secreted β-glucuronidase deconjugates estrogens [9]. These “active” deconjugated and unbound estrogens re-enter the bloodstream and subsequently act on estrogen receptors. An estrobolome enriched in enzymes favoring deconjugation, such as noted in dysbiosis, promotes reabsorption of free estrogens and thus increases total presence of systemic estrogen, potentially contributing to the risk of development of hormone-driven malignancies, such as endometrial cancer [24]. The articles included in this review demonstrated that postmenopausal women were indeed found to have increased deconjugation of estrogens in the enterohepatic circulation, expressed by significant higher levels of parent estrogens compared to estrogen metabolites (2-, 4- and 16-hydroxylated metabolites) in [42]. As estrogen metabolites have shown to display a protective effect in breast cancer, lower levels of estrogens metabolites may increase the risk of endometrial cancer as well [52]. Microbial diversity in fecal specimens was significantly associated with the ratio of estrogen metabolites to parent estrogens (E2 and E1), which grew with increasing microbiome diversity. These observational findings support the hypothesis that differences in estrogen metabolism and levels are associated with variations in gut microbial diversity [34,42]. Because all included articles compared the postmenopause to the premenopausal state, we suggest that changes in estrogen status precede changes in the gut microbiome. This is supported by the fact that diets that are enriched in isoflavones or other phyto- estrogens (estrogen metabolites) provide a source for “health beneficial” organisms in the gut microbiome, thus inducing a change in microbiome composition and function. Phyto-estrogens are thought to induce an increase of short chain fatty acid (SCFA-producing; butyrate) production and equol-producing (estrogen metabolites) organisms in the gut microbiome. SCFAs and equol are both considered bacterial metabolites exerting positive health-related effects [54,55].

Furthermore, higher systemic levels of non-ovarian estrogens, such as peripherally produced estrogen in the adipose tissue, were strongly associated with a decreased fecal microbiome richness and alpha diversity [34]. Dysbiosis resulting from increased abundance of genera that belong to Firmicutes increased these levels of non-ovarian estrogens that resulted a decreased richness in the gut microbiome. Thus, the entero-hepatic metabolism of estrogens positively influences systemic estrogen levels, which may result in an increased risk of endometrial cancer. Moreover, a relatively low abundance from the genera derived from the phyla Bacteroidetes may lead to this effect. Importantly, these associations were not present in the premenopausal state, suggesting that mainly non-ovarian estrogens are responsible for these correlations. Overexpression of the microbial enzyme β-glucuronidase was associated with larger availability of systemic deconjugated estrogens [43]. Similarly, β-glucuronidase levels were inversely correlated with both deconjugated and conjugated estrogens in feces [42] and significantly correlated with urinary estrone levels, again confirming central role of estrone and the importance of these interactions in the postmenopause and contrasting the absence of these effects premenopausally [34,42]. The relative importance of estrone may be explained by the fact that in postmenopause ovarian estrogen production has subsided.

Considering the composition of the gut microbiome in postmenopausal women, most studies, but not all, agreed that the menopausal transition increased the risk of gut dysbiosis, even independent of BMI [35,37,39]. Confounders such as diet, medication use and country of residence may have caused inconsistency in some studies. As the alteration in Firmicutes to Bacteroidetes ratio, noted in most studies, also suggests that the menopausal change enhances a disbalance leading to dysbiosis, it seems reasonable to assume the menopausal change indeed increases the risk to health-related problems [35,38,39,40]. The disbalance between the Firmicutes to Bacteroidetes phyla further enhances the before-mentioned overproduction of bacteria with ß-glucuronidase activity, confirmed by studies linking systemic estrogen levels to alterations in Firmicutes to Bacteroidetes ratio [22,56]. In a mouse study regarding colorectal cancer, it was noted that availability of 17β-estradiol (E2) reduced the Firmicutes to Bacteroidetes ratio and thereby provided protection against colorectal cancer, again suggesting an interaction between systemic estrogen (e.g., E2) levels metabolism and the Firmicutes to Bacteroidetes ratio [57]. Whether this is also the case for estrone (E1) has so far not been evaluated.

At the levels of families and genera present in the gut microbiome, the most important findings concerned a change, predominantly reduction, in SCFA organisms in menopause [35,39,41]. The SCFA-producing bacteria have local anti-inflammatory effects through induction of regulatory T-cells. By producing SCFA, they convert primary to secondary bile acids, and facilitate colonization resistance to intestinal pathogens [58]. Thus, a lower abundance may negatively impact health in postmenopausal women.

Producers of the enzyme ß-galactosidase, a negative regulator of the enzyme ß-glucuronidase, regulating hemostasis in the estrobolome, were less abundant in menopause, affecting symbiosis negatively [24]. Last, non-ovarian urine estrone levels were only strongly and significantly positively associated with the relative abundance of a number of taxa and genera from the phylum Firmicutes after menopause [42], suggesting a specific link between gut microbiome composition and this main postmenopausal estrogen. However, again, it is not clear whether in premenopause this influence is obscured by the much higher levels of ovarian estrogens and metabolites [42]. In conclusion, studies underscore an intricate relationship between the gut microbiome and systemic health, negatively affected by the menopausal changes, where changes in composition and function all point in the same direction.

Taking into account the strong relationship between the obesity and endometrial cancer, with more than half of all endometrial cancers currently attributable to obesity, our review also focused on obesity, irrespectively of menopausal status [59,60]. As gut microbiome composition differs between sexes and the gut microbiota adapts differently between the genders to, for example, dietary interventions [61], we solely included studies focusing on women. Obesity is an ever-growing threat to maintaining health, and the prevalence of obesity in women doubled during the last four decades [62].

The etiology of obesity is multifactorial, and recent studies have shown that along an imbalance between energy intake and expenditure, psychosocial and genetic characteristics, also the gut microbiome dysbiosis plays a role [63]. Consistent with previous studies, the included studies found that, overall, with increasing weight, the gut microbiome diversity reduced [40,44,45,46,47,64]. Importantly, this correlation was more pronounced in postmenopausal subjects. A metagenomic analysis comparing microbiotas belonging to identical and fraternal twins supported that reduced microbial diversity enhances caloric harvesting, suggesting that obesity and gut microbiome diversity influence each other [65]. More recent work demonstrated that individuals with low microbial gene diversity much more often were obese and that their phenotype was associated with a more marked systemic inflammation and dyslipidemia [66]. As this was a cross-sectional study, no causal effects could be described.

The increase in relative abundance of the phyla Firmicutes in obese women and women with metabolic syndrome and decreased abundance of the phyla Bacteroidetes in obesity ultimately lead to and increased Firmicutes to Bacteroidetes ratio [40,46,47,48]. The latter has been suggested a potential marker for unhealthy obesity [10,26,67]. Their corresponding ‘obesity-associated genes’ were present in Firmicutes (25%), while 42% of the lean-enriched genes were from Bacteroidetes (vs. 0% of the obesity-enriched genes). Sex-specific effects, seemingly more present in women, and fat distribution differences (e.g., male/gynoid) need to be further explored including changes at phyla and family level as highlighted by Miranda and Min [45,47]. We are only at the start of understanding the intricate interplay between adipose tissue and gut microbiome and their systemic effects. In contrast to estrogen-induced changes in the gut microbiome, the interplay between obesity and the gut microbiome is more difficult to establish because they likely influence each other as mentioned earlier. To support this even more, although it has been reported that weight changes induce differences in the gut microbiome [68], previous evidence also states that bariatric surgery did not provoke any changes in microbiome diversity, suggesting there may be genetic components that cannot be adjusted [69]. Importantly, current research about the effectiveness of a weight-loss program after endometrial cancer treatment will investigate potential gut microbiome changes that will give us more insight (*NCT03908996*; *clinicaltrial.gov* (accessed on 29 June 2021)).

In conclusion, the changes observed in obesity are associated with gut microbiome dysbiosis through reduced diversity, and increased firmicutes/bacterioidetes ratio overall parallel the changes observed after the menopausal change described above. Thus, both conditions may be additive and reinforce each other. Independently or acting together, menopausal state and female obesity are important contributors to gut microbiome dysbiosis. Therefore, it is important to establish how gut microbiome dysbiosis impacts endometrial cancer risk. The gut microbiome has been investigated in several female malignancies, and alterations have been seen in cervical cancer patients and after treatment in ovarian cancer patients [70,71,72]. In breast cancer, the role of the gut microbiome has been more established, with gut microbiome dysbiosis being associated with increased postmenopausal breast cancer risk and certain microbial commensals influencing breast cancer prognosis [43,73]. Moreover, one interesting study focused on PTEN mutated patients (among them 2 endometrial cancer patients), a frequently mutated gene in endometrioid endometrial cancer. Even in the small sample in this study, changes in gut microbiome became clear, namely, that people that suffered from cancer demonstrated a higher abundance of *Rikenellacea* (phylum Bacteroidetes; related to P13K/AKT pathway) *Eubacteriacea, Clostridia* and *Clostridiales bacerria* (phylum Fimircutes) [4]. It is plausible that these microbiome changes modulate signaling downstream in the PI3K/AKT pathway, similar to PTEN mutations and thus modify risk for both cancer and obesity. Further, the increased non-ovarian urine estrogen levels associated with fecal *Clostridia* could provide a mechanism for increased cancer risk independent of BMI [4].

Lastly, it is becoming increasingly clear that gut microbiome is closely entangled with the immune system. Gut bacteria can create a state of chronic inflammation, which is associated with tumor development [74]. Gut bacteria can upregulate the Toll-like receptors (TLR) and activate the nuclear factor kappa-light-chain-enhancer of activated B cells (NF-kB), which is important in inflammation regulation and associated with cancer. In fact, the activation of NF-kB leads to the release of IL-6, IL-12, IL-17, and IL-18 as well as the TNF-alpha, triggering chronic systemic inflammation [25]. Obese women have a significantly higher TLR5 gene expression and gut microbiome dysbiosis compared to their counterparts [48]. Pathogen-induced inflammation, however, is not limited to the site of infection as shown in a breast cancer model, where C57BL/6 ApcMin/+ mice do not develop breast tumors under specific pathogen-free conditions. However, on administration of oral *Helicobacter hepaticus*, they developed mammary carcinomas as a result of the innate immune induction through inflammation [75]. A state of chronic inflammation is also seen in obesity where the gut microbiota again seem to play a role, interacting with dietary lipids and creating inflammation in adipose tissue. Mice fed saturated fats had an increased activation of TLR, which was at least partially mediated by gut microbiota, leading to white adipose tissue inflammation [76]. Furthermore, estrogen also stimulates the production of pro-inflammatory mediators (IL-6 and TNF-alpha) [77]. The exact effect of the chronic inflammatory state through the interplay of the gut microbiome, estrogen metabolism and obesity in the development of endometrial cancer is unclear; however, the use of NSAID has been shown to decreases the risk of developing endometrial cancer in obese women, indicating a role in endometrial cancer development [78,79,80]. It is exciting to see that studies are underway investigating the effect of immunotherapy in high-grade obesity-driven endometrial cancer and the correlation between of the number of tumor infiltrating lymphocytes and microbiome profiles, both locally and in the gut (*NCT03694834*; *clinicaltrial.gov* (accessed on 29 June 2021)). Recently, in endometrial cancer, four molecular subgroups have been identified by the cancer genome atlas (TCGA) and subsequently confirmed by other groups, POLE-mutated/ultramutated (POLE), microsatellite-instable/hypermutated (MSI), copy-number-low/p53-wild-type (p53wt) and copy-number-high/p53-mutated (p53mt), in addition to the histological subtype and grade [81,82]. It will be very relevant to study gut microbiome composition differences not only related to the histopathological variables but also against these molecular subgroups related to the different genetic markers.

### Limitations

As in any review, there are several some limitations and possible biases that need to be considered. Importantly, all evidence provided in this review concerns menopausal status and female obesity and how this is associated with changes in microbiome composition and function; any evidence towards endometrial cancer is therefore at most indirect. The microbiome is modulated by a wide range of external factors, which are not consequently measured or balanced for in the included studies, such as diet, geographic location and medication use. Moreover, the number of patients per study is considered small (*n* < 71). Thus, more than anything, this review is hypothesis generating rather than drawing definitive conclusions on the microbiome–endometrial cancer association. Finally, due to all the heterogenicity in the data, we were unable to perform a meta-analysis reducing the power of the conclusions.

## 5. Conclusions

Interest is growing in the dynamic role of microbiome disturbances in human health and disease. No direct evidence is yet available to link endometrial cancer to gut microbiome (dysbiosis). Nevertheless, this review has highlighted that the gut microbiome is intrinsically linked to estrogen metabolism, menopausal state and also systemic inflammation in women. Obesity and the menopausal change may lead to a shared dysbiosis, which can be recognized by a changed gut microbial diversity and Firmicutes to Bacteroidetes ratio, making them potential hallmarks for risk stratification in endometrial cancer and possibly other hormone-dependent and obesity-driven tumors. However, inter-individual variation is large and confounders including diet and environmental factors need to be accounted for. Future studies are needed to define any causative role the gut microbiome plays in women at risk for endometrial cancer, enabling us to find targetable factors to reduce the risk of endometrial cancer development.

## Figures and Tables

**Table 1:** Characteristics and summary, of studies included for research questions 1–2 (estrogen/menopause and gut microbiome).

Study	Sample Size	Patient Characteristics	Gut Microbiota Analysis	Main Findings
Zhao et al. (2019)	*n* = 24 Premenopausal women *n* = 24 Postmenopausal women	Premenopausal: Age (yrs) 52.6 ± 6 BMI (kg/m^2^) 23.1 ± 4.5 LDL (mM) 3.0 ± 0.8 Postmenopausal Age (yrs) 53.9 ± 3.8 BMI (kg/m^2^) 23.0 ± 3.2 LDL (mM) 2.89 ± 0.83 No statistical differences.	Single-end metagenomic sequencing on BGISEQ-500 platform. Relative abundance calculation by Metaphlann2 (used by the NIH Human Microbiome Project part 2). Alpha-diversity → Shannon-index.	Alpha diversity (Shannon index): Premenopausal 1.8 Postmenopausal 1.3 (*p* 0.000005) Phyla: Firmicutes: - Premenopausal 31.6 - Postmenopausal 17.4 (*p* 0.00003) Bacteroidetes: - Premenopausal 20.1 - Postmenopausal 28.9 (*p* 0.003) Gemmatimonadetes - Premenopausal 22.5 - Postmenopausal 26.5 (*p* 0.03) Spirochetes: - Premenopausal 18.1 - Postmenopausal 30.9 (*p* 0.001) Genera: Postmenopausal state: ↓ Faecalibacterium, Alistipes, Eubacterium, and Roseburia. ↑ Bacteroides, Staphylococcus, Parabacteroides Postmenopausal: When ↑ Eubacterium rectale (stimulated by isoflavones → ability prevention dysbiosis)
Shin et al. (2019)	*n* = 9 high estrogen women (premenopausal) *n* = 8 medium estrogen women (premenopausal) *n* = 9 low estrogen women (postmenopausal)	High estrogen (>60 pg/mL) Age (yrs) 39.3 ± 3.2 BMI 28.9 ± 0.2 Medium estrogen: (5–60 pg/mL) Age (yrs) 44 ± 2 BMI (kg/m^2^) 26.9 ± 0.9 Low estrogen: (<5 pg/mL). Age (yrs) 54.9 ± 1.0 BMI (kg/m^2^) 24.9 ± 0.5 BMI not statistically different	16S V6 rRNA amplicon sequencing using QIIME. Taxonomy assigned against the Greengenes 16S rRNA gene database. Alfa diversity → Chao1 richness, Simpson evenness, Good’s coverage and Shannon diversity.	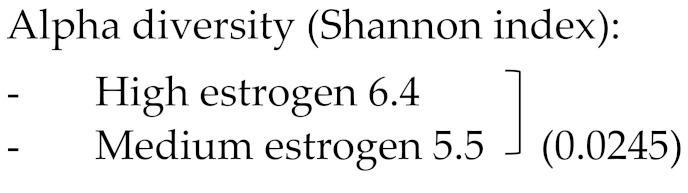 - Low estrogen 5.6 Alpha diversity (Chao1-index): - High estrogen -6900 - Low estrogen -6500 (NS) Phyla: Firmicutes: - Low estrogen 0.4 - High estrogen 0.28 (IQR; *p* < 0.05) Bacteroidetes - Low estrogen 0.58 - High estrogen 0.69 (IQR; *p* < 0.05) Firmicutes/Bacteroidetes ratio - Low estrogen 1.2 - High estrogen 0.4 (*p* < 0.05) Family within Bacteroidetes phylum: Dominant: Bacteroidaceae (61.2%), Prevotellaceae (28.6%), Rikenellaceae (3.6%) Postmenopausal vs. premenopausal ↓ Bacteroidaceae ↑ Rikenellaceae ↑ Porphyromonadaceae ↑ Odoribacteraceae Family within Firmicutes phylum: Dominant: Ruminococcaceae (42.3%), Lachnospiraceae (39.9%), Veillonellaceae (11.7%). Postmenopausal vs. premenopausal. ↓ Veillonellaceae ↑ Lachnospiraceae Specific genera for postmenopausal status: - No presence of Veillonella - Enriched in Slackia, Lactococcus, Christensenella, Dehalobacterium, Adlercreutzia, Odoribacter, and Butyricimona Link genera and serum estradiol levels. - Drop in serum estradiol through Slackia (r = −0.4; *p* 0.033) Butyricimonas (r = −0.4; *p* 0.046)
Zhu et al. (2018)	*n* = 25 premenopausal women *n* = 46 postmenopausal women (breast cancer patients excluded)	Premenopausal Age (yrs) 35.5 ± 6.0 BMI (kg/m^2^) 23.0 ± 2.0 Postmenopausal Age (yrs) 56.9 ± 6.4 BMI (kg/m^2^) 24.0 ± 2.5 BMI not statistically different	Illumina DNA sequencing. Taxonomy calculated against the integrated reference catalog of the human gut microbiome (IGC) by bowtie2 Alfa-diversity → Shannon index, Chao index	Alpha diversity (Shannon index) Premenopausal 3.1 Postmenopausal 3.2 (*p*-value not calculated) Alpha diversity (Chao1 index) Premenopausal −430 Postmenopausal −415 (*p*-value not calculated) Alpha diversity (OTU) Premenopausal −400 Postmenopausal −390 (*p*-value not calculated) Link genera and serum estradiol levels. - Shewanella putrefaciens (Spearman rho = 0.379; *p* = 0.025) - Erwinia amylovora (Spearman rho = 0.351; *p* = 0.039)
Santos-Marcos et al. (2018)	*n* = 17 premenopausal women *n* = 20 postmenopausal women	Premenopausal Age (yrs) 46.1 ± 0.8 BMI (kg/m^2^) 26.3 ± 1.5 LDL (mg/dL) 119 ± 7 Postmenopausal Age (yrs) 55.6 ± 0.6 BMI (kg/m^2^) 28.9 ± 1.3 LDL (mg/dL) 137 ± 7	Sequencing the V1–V2 microbial 16S rRNA gene on the Illumina MiSeq Taxonomy assigned against Greengenes v13-8 database	Phyla: Firmicutes - Premenopausal 44.1% - Postmenopausal 50.7% Bacteroidetes - Premenopausal 48% - Postmenopausal 43.4% Firmicutes/Bacteroidetes ratio - Premenopausal 1.2 - Postmenopausal 2.1 (*p* 0.01) Actinobacteria phylum - Premenopausal - Postmenopausal (*p* 0.001) Genus: Premenopausal ↑ Parabacteroides (*p* = 0.002) ↑ Prevotella (*p* < 0.001) ↑ Bilophila Postmenopausal ↑ Lachnospira (*p* = 0.047) ↑ Roseburia (*p* = 0.003) Link gut microbiome and estradiol levels: Positively correlated: - Class Gammaproteobacteria (R = 0.575, *p* = 0.013) - Family from Mixococcales (R = 0.521, *p* = 0.039, respectively) Negatively correlated - Family Prevotellaceae (R = −0.523 *p* = 0.018) Immunology: TNF-alfa (pg/mL) Premenopausal 0.26 (±0.05) Postmenopausal 0.38 (±0.06; NS) IL-6 (pg/mL) Premenopausal 1.25 (±0.15) Postmenopausal 1.75 (±0.25; *p* 0.036) MCP-1 (pg/mL) Premenopausal 72 (±4) Postmenopausal 94 (±7; *p* 0.045)
Choi et al. (2017) Animal study	*n* = 3 SHAM mice *n* = 5 ovariectomized mice (OVX)	SHAM Weight (g) 29.96 ± 2.13 LDL (mg/dL) 30.9 ± 5.1 OVX Weight (g) 41.44 ± 1.52 LDL (mg/dL) 45.1 ± 9.1 Weight significantly different	V3-V4 16S rRNA amplification following the 16S Metagenomic Sequencing Library Preparation guide by Illumina. Gene-enrichment and functional annotation analysis performed using gene ontology and KEGG pathway analysis.	Alpha diversity (Shannon index) - SHAM 3.3 - SHAM-HF (significant reduction) - OVX 2.4 (significant reduction) - OVX-HF no difference controls Phyla Firmicutes - SHAM mice: 20% - OVX mice: 90% Bacteroidetes - HAM mice: 78% - OVX mice: 2% Actinobacteria - Increase in abundance in OVX mice Genus and species SHAM ↑ Prevotella (*p* 0.036) ↑ Bacteroides (*p* 0.036) ↑ Bacteroidales (*p* 0.036) OVX ↑ Lactobacillus species (*p* 0.049) ↑ Bifidobacterium animalis (exclusively present in this group *p* 0.043) Link gut microbiome and estradiol - Gene expression of estrogen signaling pathways differed significantly between OVX and SHAM Differences in the microbiome caused by ovariectomy similar to those caused by the high-fat diet
Zhang et al. (2017) Animal study	*n* = 6 SHAM rats *n* = 12 OVX - *n* = 6 OVX vehicle - *n* + 6 with curcumin	All groups: Virgin Wistar rats Age (yrs) 0.5 Weight: 310 ± 20.0 g (OVX rats significantly higher weight)	The estradiol concentration in the serum detected through electrochemiluminescence immunoassay (ECLIA)	Alpha diversity (Shannon index) - SHAM 7.0 - OVX 7.16 Phyla - Firmicutes - SHAM: 0.26 ± 0.05 - OVX: 0.53 ± 0.07 Bacteroidetes - SHAM 0.66 ± 0.07 - OVX: 0.32 ± 0.11 Genus Incertae_Sedis - SHAM: 0.04 ± 0.00 - OVX: 0.10 ± 0.01 (*p* 0) Heliobacter - SHAM 0.03 ± 0.01 - OVX: 0.15 ± 0.051 (*p* 0.002) Anaerovorax - SHAM 0.000233 ± 0.00 - OVX 0.001323 ± 0.00 (*p* 0.02) Anaerotruncus - 0.003101 ± 0.00 - 0.006812 ± 0.00 (*p* 0.04)
Fuhrman et al. (2014)	*n* = 6 postmenopausal women (acting as their own controls)	Postmenopausal Age (yrs) 60.2 ± 3.2 BMI (kg/m^2^) 27.3 ± 5.4	Pyrosequencing V1–V2 16S rRNA amplicons, QIIME: Ribosomal Data Project Bayesian classifier.	Alpha diversity (Shannon index) - Postmenopausal 6.6 ± 6.6 Phyla Firmicutes - Postmenopausal 63.5% (IQR 48.4–73.1%) Bacteroidetes - Postmenopausal 35.6% (IQR 26.4–50.4%) Link gut microbiome to estradiol levels. Positive correlation ratio of estrogen metabolites to parent estrogen: - Order clostridiales (R0.32; *p* 0.02) - Family Ruminococcaceae (R0.37; *p* 0.05). Negative correlation ratio of estrogen metabolites to parent estrogen: - Genus bacteroides (R-0.3; *p* 0.03). Urinary estrogen (pM/mg creatinin) Postmenopausal women 28.1 (±17.8) Parent estrogen (estrone and estradiol 32 % of total EM’s) 2-, 4- and 16-hydroxilated metabolites represented 29%, 3% and 35%)
Flores et al. (2012)	*n* = 19 premenopausal women *n* = 7 postmenopausal women *n* = 22 age matched men (55 yrs and older)	Average BMI 26	In feces, β-glucuronidase and β-glucosidase activities were determined by real-time kinetics, and microbiome diversity and taxonomy were estimated by pyrosequencing 16S rRNA amplicons.	Urinary estrogen (pM/mg creatinine): men 82.6 premenopausal women 68.7 postmenopausal women 155.1 Levels non-ovarian estrogens Premenopausal - No association with alpha diversity. Postmenopausal - Strongly associated with fecal microbiome richness and alpha diversity Non-ovarian urine estrogen (estrone) levels strongly and significantly associated with Postmenopausal - Taxa Clostridia (Firmicutes,) - 3 genera from family Ruminococcaceae (β = 0.57 to 0.70, *p* = 0.03 to 0.002 Premenopausal: - Correlation premenopausal estrogens with abundance Clostridiales Firmicutes almost nil (β = −0.10, *p* = 0.55) Fecal β-glucuronidase activity: Postmenopausal - Significantly correlated with urine estrone level (R = 0.36, *p* = 0.04) but not with total urine estrogens (R = 0.24, *p* = 0.19), estradiol (R = 0.16, *p* = 0.38), or EM Premenopausal - Urine estrogens not correlated with either β-glucuronidase or β-glucosidase activity. Activity of the control enzyme, β-glucosidase, was not related to total urine estrogens (R = 0.12) or to any of the parent estrogens or EM Fecal estrogens in postmenopause: - Deconjugated estrogens inversely correlated with total estrogen levels in urine; especially strong for deconjugated fecal estrone (R = −0.50, *p* = 0.005) - Conjugated not correlated with urinary estrogen levels - Fecal β-glucuronidase activity inversely correlated with both deconjugated and conjugated estrogens in feces (*p* ≤ 0.01 for all except 16-epiestriol) - Shannon index and OTU species were strongly and significantly associated with lower levels of conjugated and especially deconjugated estrogens in feces

**Table 2:** Characteristics and summary, of studies included for research question 3.

(a) Obesity in Women and Gut Microbiome				
Study	Sample Size	Patient Characteristics	Gut Microbiota Analysis	Main Findings
Menni et al. (2016)	*n* = 544 women with weight loss: BMI from 25.4 to 24.4 (group 1) *n* = 544 women with little weight gain: BMI from 24 to 25.2 (group 2) *n* = 544 women with heavy weight gain BMI from 25.4 to 28.8 (group 3)	Group 1 Age (yrs) 49.91 ± 9.49 Group 2 Age (yrs) 50.11 ± 5.54 Group 3 Age (yrs) 49.25 ± 8.48 All groups 15% smokers, further no exclusions.	V4 region of the 16S ribosomal RNA gene was amplified and sequenced on Illumina. De novo OTU clustering was carried across all reads using Sumaclust within QIIME 1.9.0. Alpha diversities → Shannon index, OTU counts.	Alpha diversity (Shannon index): Group 1 (weight loss) 5.21 Group 2 (weight gain) 5.19 Group 3 (heavy weight gain) 5.07 (*p* < 0.05) Alpha diversity (OTU): Group 1 346.3 Group 2 348 Group 3 331.8 (*p* < 0.05) Family Bacteriodes - Positive correlation weight gain (OR = 1.18 (0.04) *p* = 0.002). Negative correlation microbiome diversity Ruminococcaceae (firmicutes phyla) - Nominally protective of weight gain (OR = 0.89 (0.05), *p* = 0.038)
Chavez-Carbajal et al. (2019)	*n* = 25 control women *n* = 17 obese women *n* = 25 obese women with metabolic syndrome	Controls Age (yrs) 23.3 ± 3.1 BMI (kg/m^2^) 21.4 ± 1.9 Obesity Age (yrs) 28.8 ± 8.4 BMI (kg/m^2^) 34.8 ± 6.1 Obesity + metabolic syndrome (ms) Age (yrs) 40.5 ±10.3 BMI (kg/m^2^) 35.8 ± 5.1 Only women to avoid gender bias Controls significant different in age and bmi from other 2 groups	V3 region of the 16S rDNA Amplicon PCR amplification using PCR GeneAmp System 2700 Thermal Cycler. Determine with an open reference the OTUs and using a 97% similarity using QIIME pipeline (v1.9.0) and Geengenes database v13.8. Alpha diversity → Observed Species, Chao1, Shannon, Simpson.	Alpha diversity (Shannon index) Controls 4.9 Obesity 5.23 Obesity + MS 5.15 Dominant phyla in all groups: Firmicutes, Bacteroidetes, Proteobacteria, and Actinobacteria Phyla Frimicutes - Controls 57.0% - Obesity 73.0% - Obesity + MS 73.3% (*p* = 0.003) Bacteroidetes - Controls 36.2% - Obesity 22.5% - Obesity + MS 23.4% (*p* = 0.7) Firmicutes to Bacteroidetes ratio - Controls 1.57 - Obesity 3.24 - Obesity + MS 3.13 Genus Obesity and obesity + MS ↓ Bacteroides, compared to controls (*p* < 0.0001) Faecalibacterium (phyla firmicutes) Controls 0.55% ↑ Obesity 1.2% ↑ Obesity + MS 1.2% (*p* = 0.0003) Roseburia (phyla firmicutes) Controls 0.89% ↑ Obesity 2.72% ↑ Obesity + MS 2.14% (*p* = 0.0002) Lachnospira (phyla firmicutes) Controls 0.99% ↑ Obesity 3.24% ↑ Obesity + MS 3.79% (*p* < 0.0001) Coprococcus, (phyla firmicutes) Controls 2.18% ↑ Obesity 4.55% ↑ Obesity + MS 4.51% (*p* = 0.0002) family Erysipelotrichaceae (firmicutes) Controls 1.74% ↓ Obesity 0.38% ↓ Obesity + MS 0.36% (*p* < 0.0001)
Miranda er al. (2017) Observational study	*n* = 31 controls *n* = 32 normal BMI but high body fat percentage. *n* = 33 obesity	Controls Age (yrs) 16.3 ± 0.8 Gynoid fat (%) 34.5 (30.6–36.7) High body fat Age (yrs) 16.5 ± 0.9 Gynoid fat (%) 39.7 (37.9–46.9) Obesity Age (yrs) 16.2 ±1.3 Gynoid fat (%) 48.0 (45.5–54.1)	RT-qPCR to analyze microbiota CFX96 Touch™ detection system (Bio-Rad, Hercules, CA, USA) Alfa diversity → Shannon index	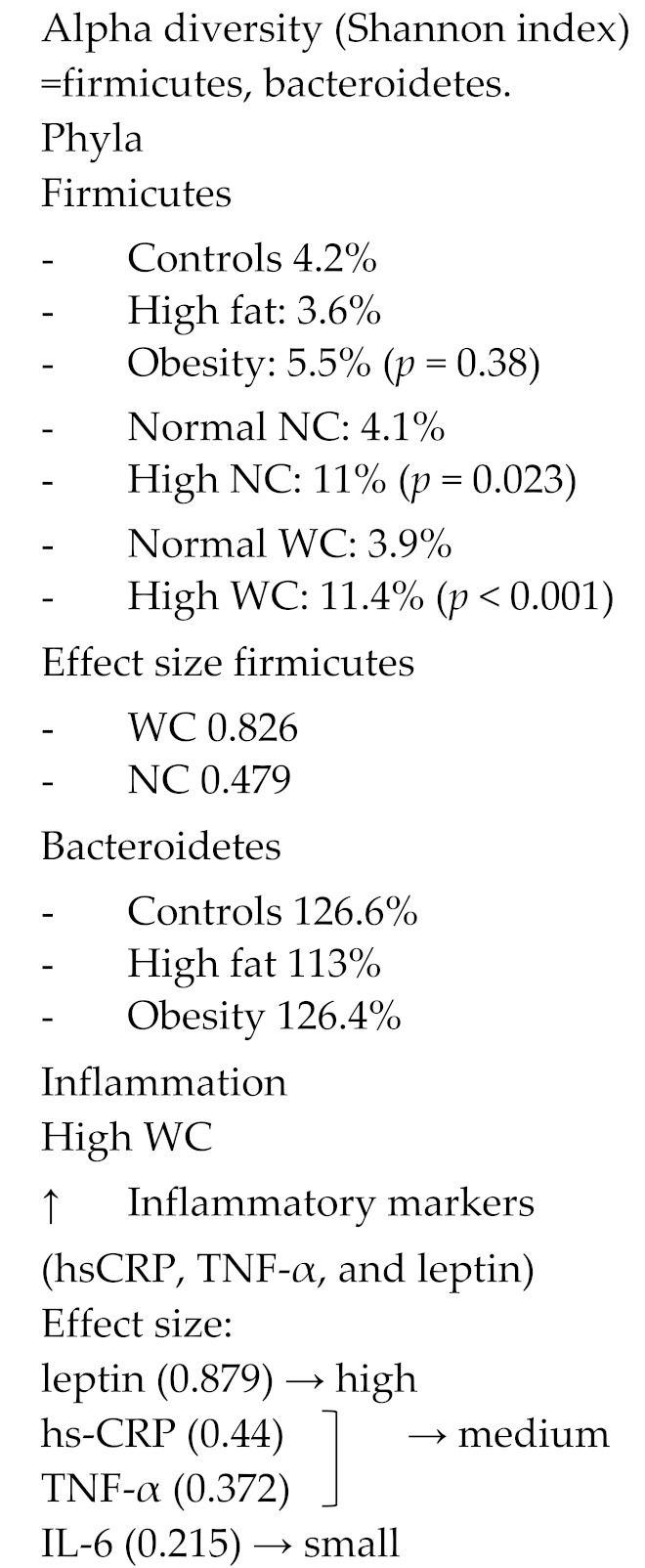
Pekkala et al. (2015)	*n* = 4 women with high TLR gene expression (BMI 31) *n* = 4 women with low TLR gene expression (BMI 28)	High TLR gene expression Age (yrs) 35.5 ± 6.0 BMI (kg/m^2^) 31 ± 2.0 Low TLR gene expression Age (yrs) 56.9 ± 6.4 BMI (kg/m^2^) 28 ± 2.5 BMI significantly higher in High TLR group.	Real-time PCR analysis was performed using in-house designed primers, iQ SYBR Supermix and CFX96 TM Real-time PCR Detection System (Bio-Rad Laboratories) Real-time PCR analysis was performed using in-house designed primers, iQ SYBR Supermix and CFX96 TM Real-time PCR Detection System (Bio-Rad Laboratories) Real-time PCR analysis was performed using in-house designed primers, iQ SYBR Supermix and CFX96 TM Real-time PCR Detection System (Bio-Rad Laboratories) RNA extraction and rt-PCR analysis using in-house designed primers.	Alpha diversity High TLR group: significant dysbiosis. Phyla Firmicutes to Bacteroidetes ratio - Low TLR 5% (*p* < 0.05) - High TLR 15% Cluster - High TLR: ↓ flagellated clostridium (*p* = 0.029) Genus - High TLR: ↓ 20% Bifidobacterium
Ott et al. (2018)	*n* = 20 women (own controls) *n* = 20 after diet *n* = 20 14 days after diet	Women Age (yrs) 46.8 ± 11.5 Before diet BMI (kg/m^2^) 34.9 ± 3.8 After diet BMI (kg/m^2^) 32.5 ± 3.5 14 dys after diet BMI (kg/m^2^) 32.6 ± 3.8	16 S rRNA gene amplicons were sequenced in paired-end modus (PE275) using a MiSeq system (Illumina)	Alpha diversity No differences Phyla Protobacteria ↓ after decrease BMI (*p* < 0.05) Firmicutes (after decrease BMI) ↓ Tree OTUs from family Lachnospiraceae ↓ Ruminococcaceae Actinobacteria (after decrease BMI) ↑ Bifdobacteriaceas
Choi et al. (2017) Animal study	*n* = 3 SHAM mice *n* = 3 SHAM-HF *n* = 5 ovariectomized mice (OVX) *n* = 5 OVX-HF	SHAM Weight (g) 29.96 ± 2.13 LDL (mg/dL) 30.9 ± 5.1 SHAM-HF Weight (g) 53.13 ± 3.88 LDL (mg/dL) 78 ± 4.4 OVX Weight (g) 41.44 ± 1.52 LDL (mg/dL) 45.1 ± 9.1 OVX-HF Weight (g) 57.54 ± 3.84 LDL (mg/dL) 95.7 ± 12.3 Weight significantly different	V3-V4 16S rRNA amplification following the 16S Metagenomic Sequencing Library Preparation guide by Illumina. Gene-enrichment and functional annotation analysis performed using gene ontology and KEGG pathway analysis.	Alpha diversity (Shannon index) - SHAM 3.3 - SHAM-HF 2.4 (significant reduction) - OVX 2.4 (significant reduction) - OVX-HF 2.7 Phyla Firmicutes - SHAM mice: 20% - OVX mice: 90% Bacteroidetes - SHAM mice: 78% - OVX mice: 2% Verrucomicrobia Proteobacteria - Increase in OVX-HF mice Genus and species SHAM ↑ Prevotella (*p* 0.036) ↑ Bacteroides (*p* 0.036) ↑ Bacteroidales (*p* 0.036) SHAM-HF ↑ Lactobacillus species ↑ Clostridiales Link gut microbiome and estradiol - estrogen signaling pathways not different in the OVX-HF vs. SHAM-HF Microbiome host interaction in OVX - OVX Lactobacillus species interaction metabolic pathways, antibiotic biosynthesis pathways, FoxO signaling pathway, glycerophospholipid metabolism pathway, and steroid hormone biosynthesis pathway. Akkermansia muciniphila related to - Pik3ca and Lgf1 → estrogen signaling pathway and ovarian steroidogenesis pathway - Cyp26b1, Nnmt, Pnpla3, and Ptgds, → metabolic pathways. - OVX-HF Ruminococcus, Dorea species, and A. muciniphila correlation with metabolic pathway, MAPK signaling pathway, AMPK signaling pathway, and FoxO signaling pathway.
**(b) Obesity and Gut Microbiome: Sex Differences**				
**Study**	**Sample Size**	**Patient Characteristics**	**Gut Microbiota Analysis**	**Main Findings**
Haro et al. (2016)	*n* = 39 men *n* = 13 men < BMI 30 *n* = 13 BMI 30–33 *n* = 13 men BMI > 33 *n* = 36 women *n* = 13 BMI < 30 *n* = 10 BMI 30–33 *n* = 23 BMI > 33	Men BMI < 30 Age (yrs) 63.2 ± 2.0 BMI (kg/m^2^) 27.6 ± 0.6 LDL (mg/dL) 76.6 ± 4.2 BMI 30–33 Age (yrs) 58.9 ± 2.4 BMI (kg/m^2^) 31.4 ± 0.3 LDL (mg/dL) 95.3 ± 6.0 BMI > 33 Age (yrs) 61.3 ± 2.2 BMI (kg/m^2^) 35.3 ± 0.7 LDL (mg/dL) 87.8 ± 2.1 Women BMI < 30 Age (yrs) 60.1 ± 2.6 BMI (kg/m^2^) 27.0 ± 0.8 LDL (mg/dL) 94.2 ± 9.4 BMI 30–33 Age (yrs) 62.4 ±2.3 BMI (kg/m^2^) 31.4 ± 0.3 LDL (mg/dL) 87.1 ± 7.6 BMI > 33 Age (yrs) 58.9 ± 2.3 BMI (kg/m^2^) 36.7 ± 1.4 LDL (mg/dL) 80.4 ± 4.4	Sequencing V4 16S microbial rRNA on the Illumina MiSeq. Taxonomy assigned to OTUs against the Greengenes v13-8 preclustered at 97% identity. Alpha diversities → observed OTU counts, Shannon, Simpson.	Alpha diversity similar men and women and comparing BMI Phyla Firmicutes to Bacteroidetes ratio - BMI < 33: Men higher ratio - BMI > 33: Women higher ratio (*p* = 0.018) Genus Women BMI > 33 ↑ Bilophila (*p* = 0.002) ↑ Veillonella (*p* = 0.001) Men BMI > 33 ↑ Methanobrevibacter (*p* = 0.002) Bacterial species Women BMI > 33 ↑ Bacteroides caccae (*p* = 0.009) Men BMI > 33 ↑ Bacteroides plebeius (*p* = 0.001) ↑ Coprococcus catus
Min et al. (2019)	*n* = 116 women *n* = 96 men	Women Age (yrs) 50.7 ± 14.1 BMI (kg/m^2^) 23.0 ± 3.0 Gynoid fat 15.9 ± 3.0 Android fat 12.5 ± 1.2 LDL (mmol/L) 2.7 ± 0.7 Men Age (yrs) 50.7 ± 14.5 BMI (kg/m^2^) 23.6 ± 3.0 Gynoid fat 17.7 ± 3.0 (*p* < 0.005) Android fat 9.9 ± 1.4 (*p* < 0.005) LDL (mmol/L) 2.8 ± 0.7	16S rRNA V4 region sequencing The denoised sequences are mapped to the GreenGenes reference database43. Taxonomy is assigned at 97% identity. Alfa diversity → Shannon index	Alpha diversity potential negative association between gynoid fat ratio and microbiome abundance in both sexes. In women compared to men different taxa responsible for relation between fat distribution and diversity. Gynoid fat ratio positive correlation Women: - Provotellaceae family (effect size 9.6) - Ruminococcaceae family Men: - Lachnospiraceae family - Clostridium_XlVa (effect size 10) Gynoid fat ratio negative correlation - Bacteroidaceae family, Bacteroides genus (effect size of −24.2) - Ruminococcaceae family No taxa associated with android fat ratio.

**Table 3:** Risk of bias, organized according to study design.

Case-Control	*NOS Scale*			
	Selection	Comparibilty	Exposure	
				
Byrd et al.	**	*	***	
Zhao et al.	**	**	***	
Shin et al.	*	**	***	
Zhu et al.	**	**	**	
Santos-Marcos et al.	****	**	***	
Menni et al.	***	**	***	
Chavez et al.	**	**	***	
Miranda et al.	***	**	**	
Pekkala et al.	****	**	**	
Haro et al.	***	**	***	
Min et al.	***	*	***	
				
** Cohort **	**NOS Scale**			
	Selection	Comparability	Outcome	
				
Ott et al.	**	**	***	
				
**Cross-Sectional**	**AXIS**			
	Intro	Methods	Results	Discussion/Ethics
				
Fuhrman et al.	1/1	7/11	4/5	4/4
Flores et al.	1/1	6/11	4/5	4/4
				
** Animal **	**SYRCLE’s**	**Bias Tool**		
	Selection/Performance	Detection	Attrition	Reporting
Choi et al.	2/5	0/2	1/1	1/1
Zhang et al.	0/5	0/2	1/1	1/1

Risk of Bias table, graded according to NOS scale for assessing the quality of non-randomized studies for cohort studies and case-control studies in systematic reviews and meta-analyses. Further graded according to AXIS for cross-sectional studies and graded using the SYRCLE’s bias tool for animal studies.

## Data Availability

Not applicable.

## Supplementary Materials

The following are available online at https://www.mdpi.com/article/10.3390/jcm10132916/s1. Supplemental File S1. Search strategies. Supplemental file S2. Risk of Bias tools.
