# Supplementary material for: How the Gut Microbiome Links to Menopause and Obesity, with Possible Implications for Endometrial Cancer Development
Source: J Clin Med. 2021 Jun 29;10(13):2916. doi: 10.3390/jcm10132916 (PMC8268108; doi:10.3390/jcm10132916)
Supplement: Supplementary file 1 [file jcm-10-02916-s001.zip › jcm-1268628-supplementary.pdf]

Supplemental File S1. Search strategies

Search strategy research question 1

1. "Estrogen"[Mesh]

2. "Estradiol"[Mesh]

3. “Seks steroid hormones” [Mesh]

4. 1 OR 2 OR 3

5. "Gut microbiome"[Mesh]

6. "Intestinal microbiome"[Mesh]

7. "Fecal microbiome” [Mesh]

8. “Enteric microbiome” [Mesh]

9. “Gastrointestinal microbioma” [Mesh]

10. "Gut microbiota"[Mesh]

11. "Intestinal microbiota"[Mesh]

12. "Fecal microbiota” [Mesh]

13. “Enteric microbiota” [Mesh]

14. “Gastrointestinal microbiota” [Mesh]

15. "Gut microflora"[Mesh]

16. "Intestinal microflora"[Mesh]

17. "Fecal microflora” [Mesh]

18. “Enteric microflora” [Mesh]

19. “Gastrointestinal microflora” [Mesh]

20. "Gut dysbiosis"[Mesh]

21. "Intestinal dysbiosis "[Mesh]

22. "Fecal dysbiosis” [Mesh]

23. “Enteric dysbiosis” [Mesh]

24. “Gastrointestinal dysbiosis” [Mesh]

25. 7 OR 8 OR 9 OR 10 OR 11 OR 12 OR 13 OR 14 OR 15 OR 16 OR 17 OR 18 OR 19 OR 20 OR 21 OR 22 OR 23 OR 24

26. “English”[Lang]

27. “review”[publicationtype]

28. 6 AND 25 AND 26 NOT 27

Search strategy research question 2

1. "Menopause"[Mesh]

2. "Postmenopause"[Mesh]

3. “Postmenopausal women” [Mesh]

4. “Postmenopausal” [Mesh]

5. 1 OR 2 OR 3 OR 4

6. "Gut microbiome"[Mesh]

7. "Intestinal microbiome"[Mesh]

8. "Fecal microbiome” [Mesh]

9. “Enteric microbiome” [Mesh]

10. “Gastrointestinal microbioma” [Mesh]

11. "Gut microbiota"[Mesh]

12. "Intestinal microbiota"[Mesh]

13. "Fecal microbiota” [Mesh]

14. “Enteric microbiota” [Mesh]

15. “Gastrointestinal microbiota” [Mesh]

16. "Gut microflora"[Mesh]

17. "Intestinal microflora"[Mesh]

18. "Fecal microflora” [Mesh]

19. “Enteric microflora” [Mesh]

20. “Gastrointestinal microflora” [Mesh]

21. "Gut dysbiosis"[Mesh]

22. "Intestinal dysbiosis "[Mesh]

23. "Fecal dysbiosis” [Mesh]

24. “Enteric dysbiosis” [Mesh]

25. “Gastrointestinal dysbiosis” [Mesh]

26. 6 OR 7 OR 8 OR 9 OR 10 OR 11 OR 12 OR 13 OR 14 OR 15 OR 16 OR 17 OR 18 OR 19 OR 20 OR 21 OR 22 OR 23 OR 24 OR 25

27. “English”[Lang]

28. “review”[Publication type]

29. 5 AND 26 AND 27 NOT 28

<u>Search strategy research question 3</u>

1. "Overweight"[Mesh]

2. "Obese"[Mesh] OR “Obesity”

3. “Obese women” [Mesh]

4. “Overweight women” [Mesh]

5. 1 OR 2 OR 3 OR 4

6. "Gut microbiome"[Mesh]

7. "Intestinal microbiome"[Mesh]

8. "Fecal microbiome” [Mesh]

9. “Enteric microbiome” [Mesh]

10. “Gastrointestinal microbioma” [Mesh]

11. "Gut microbiota"[Mesh]

12. "Intestinal microbiota"[Mesh]

13. "Fecal microbiota” [Mesh]

14. “Enteric microbiota” [Mesh]

15. “Gastrointestinal microbiota” [Mesh]

16. "Gut microflora"[Mesh]

17. "Intestinal microflora"[Mesh]

18. "Fecal microflora” [Mesh]

19. “Enteric microflora” [Mesh]

20. “Gastrointestinal microflora” [Mesh]

21. "Gut dysbiosis"[Mesh]

22. "Intestinal dysbiosis "[Mesh]

23. "Fecal dysbiosis” [Mesh]

24. “Enteric dysbiosis” [Mesh]

25. “Gastrointestinal dysbiosis” [Mesh]

26. 6 OR 7 OR 8 OR 9 OR 10 OR 11 OR 12 OR 13 OR 14 OR 15 OR 16 OR 17 OR 18 OR 19 OR 20 OR 21 OR 22 OR 23 OR 24 OR 25

27. “English”[Lang]

28. “review”[Publication type]

29. 5 AND 26 AND 27 NOT 28

Search strategy research question 4 (to confirm there is no available literature about the direct link between the gut microbiome and endometrial cancer)

Full electronic search strategy used in PubMed:

1. "Endometrial Neoplasms"[Mesh]

2. "Uterine Neoplasms"[Mesh]

3. “Endometrial cancer” [Mesh]

4. “Uterine cancer” [Mesh]

5. “endometrial hyperplasia” [Mesh]

6. 1 OR 2 OR 3 OR 4 OR 5

7 "Gut microbiome"[Mesh]

8. "Intestinal microbiome"[Mesh]

9. "Fecal microbiome” [Mesh]

10. “Enteric microbiome” [Mesh]

11. “Gastrointestinal microbiome” [Mesh]

12. "Gut microbiota"[Mesh]

13. "Intestinal microbiota"[Mesh]

14. "Fecal microbiota” [Mesh]

15. “Enteric microbiota” [Mesh]

16. “Gastrointestinal microbiota” [Mesh]

17. "Gut microflora"[Mesh]

18. "Intestinal microflora"[Mesh]

19. "Fecal microflora” [Mesh]

20. “Enteric microflora” [Mesh]

21. “Gastrointestinal microflora” [Mesh]

22. "Gut dysbiosis"[Mesh]

Supplemental file S2. Risk of Bias tools

Newcastle-Ottawa assessment scale (NOS)

These two versions of the NOS assessment scale for case-control and cohort studies were retrieved from the following website: http://www.ohri.ca/programs/clinical_epidemiology/nosgen.pdf.

Case-Control studies

Selection

1) Is the case definition adequate?
   a) yes, with independent validation ✱
   b) yes, eg record linkage or based on self reports
   c) no description

2) Representativeness of the cases
   a) consecutive or obviously representative series of cases ✱
   b) potential for selection biases or not stated

3) Selection of Controls
   a) community controls ✱
   b) hospital controls
   c) no description

4) Definition of Controls
   a) no history of disease (endpoint) ✱
   b) no description of source

Comparability

1) Comparability of cases and controls on the basis of the design or analysis
   a) study controls for _______________ (Select the most important factor.) ✱
   b) study controls for any additional factor ✱ (This criteria could be modified to indicate specific control for a second important factor.)

Exposure

1) Ascertainment of exposure
   a) secure record (eg surgical records) ✱
   b) structured interview where blind to case/control status ✱
   c) interview not blinded to case/control status
   d) written self report or medical record only
   e) no description

2) Same method of ascertainment for cases and controls
   a) yes ✱
   b) no

3) Non-Response rate
   a) same rate for both groups ✱
   b) non respondents described
   c) rate different and no designation

Cohort studies

Selection

1) Representativeness of the exposed cohort
    a) Truly representative ***(one star)***
    b) Somewhat representative ***(one star)***
    c) Selected group
    d) No description of the derivation of the cohort
2) Selection of the non-exposed cohort
    a) Drawn from the same community as the exposed cohort ***(one star)***
    b) Drawn from a different source
    c) No description of the derivation of the non exposed cohort
3) Ascertainment of exposure
    a) Secure record (e.g., surgical record) ***(one star)***
    b) Structured interview ***(one star)***
    c) Written self report
    d) No description
    e) Other
4) Demonstration that outcome of interest was not present at start of study
    a) Yes ***(one star)***
    b) No

Comparability

1) Comparability of cohorts on the basis of the design or analysis controlled for confounders
    a) The study controls for age, sex and marital status ***(one star)***
    b) Study controls for other factors (list) ________________________________ ***(one star)***
    c) Cohorts are not comparable on the basis of the design or analysis controlled for confounders

Outcome

1) Assessment of outcome
    a) Independent blind assessment ***(one star)***
    b) Record linkage ***(one star)***
    c) Self report
    d) No description
    e) Other
2) Was follow-up long enough for outcomes to occur
    a) Yes ***(one star)***
    b) No

Indicate the median duration of follow-up and a brief rationale for the assessment above:____________________

3) Adequacy of follow-up of cohorts
    a) Complete follow up- all subject accounted for ***(one star)***
    b) Subjects lost to follow up unlikely to introduce bias- number lost less than or equal to 20% or description of those lost suggested no different from those followed. ***(one star)***
    c) Follow up rate less than 80% and no description of those lost
    d) No statement

AXIS tool for cross-sectional studies

This tool is the final AXIS tool following consensus by all components from the Delphi panel. It was retrieved from*: Downes et al, BMJ OPEN 2016. Pubmed ID:* *27932337*

[illegible]

		Yes	No	Do not know/ comment
Introduction				
1	Were the aims/objectives of the study clear?			
Methods				
2	Was the study design appropriate for the stated aim(s)?			
3	Was the sample size justified?			
4	Was the target/reference population clearly defined? (Is it clear who the research was about?)			
5	Was the sample frame taken from an appropriate population base so that it closely represented the target/reference population under investigation?			
6	Was the selection process likely to select subjects/participants that were representative of the target/reference population under investigation?			
7	Were measures undertaken to address and categorise non-responders?			
8	Were the risk factor and outcome variables measured appropriate to the aims of the study?			
9	Were the risk factor and outcome variables measured correctly using instruments/ measurements that had been trialled, piloted or published previously?			
10	Is it clear what was used to determined statistical significance and/or precision estimates? (eg, p values, CIs)			
11	Were the methods (including statistical methods) sufficiently described to enable them to be repeated?			
Results				
12	Were the basic data adequately described?			
13	Does the response rate raise concerns about non-response bias?			
14	If appropriate, was information about non-responders described?			
15	Were the results internally consistent?			
16	Were the results for the analyses described in the methods, presented?			
Discussion				
17	Were the authors' discussions and conclusions justified by the results?			
18	Were the limitations of the study discussed?			
Other				
19	Were there any funding sources or conflicts of interest that may affect the authors' interpretation of the results?			
20	Was ethical approval or consent of participants attained?			

SYRCLE's risk of bias tool for animal studies

This risk of bias tool designed for animal studies was retrieved from: *Hooijmans et al. BMC open. 2014*
Pubmed ID: 24667063

Table 2 SYRCLE's tool for assessing risk of bias

Item	Type of bias	Domain	Description of domain	Review authors judgment
1	Selection bias	Sequence generation	Describe the methods used, if any, to generate the allocation sequence in sufficient detail to allow an assessment whether it should produce comparable groups.	Was the allocation sequence adequately generated and applied? (*)
2	Selection bias	Baseline characteristics	Describe all the possible prognostic factors or animal characteristics, if any, that are compared in order to judge whether or not intervention and control groups were similar at the start of the experiment.	Were the groups similar at baseline or were they adjusted for confounders in the analysis?
3	Selection bias	Allocation concealment	Describe the method used to conceal the allocation sequence in sufficient detail to determine whether intervention allocations could have been foreseen before or during enrolment.	Was the allocation adequately concealed? (*)
4	Performance bias	Random housing	Describe all measures used, if any, to house the animals randomly within the animal room.	Were the animals randomly housed during the experiment?
5	Performance bias	Blinding	Describe all measures used, if any, to blind trial caregivers and researchers from knowing which intervention each animal received. Provide any information relating to whether the intended blinding was effective.	Were the caregivers and/or investigators blinded from knowledge which intervention each animal received during the experiment?
6	Detection bias	Random outcome assessment	Describe whether or not animals were selected at random for outcome assessment, and which methods to select the animals, if any, were used.	Were animals selected at random for outcome assessment?
7	Detection bias	Blinding	Describe all measures used, if any, to blind outcome assessors from knowing which intervention each animal received. Provide any information relating to whether the intended blinding was effective.	Was the outcome assessor blinded?
8	Attrition bias	Incomplete outcome data	Describe the completeness of outcome data for each main outcome, including attrition and exclusions from the analysis. State whether attrition and exclusions were reported, the numbers in each intervention group (compared with total randomized animals), reasons for attrition or exclusions, and any re-inclusions in analyses for the review.	Were incomplete outcome data adequately addressed? (*)
9	Reporting bias	Selective outcome reporting	State how selective outcome reporting was examined and what was found.	Are reports of the study free of selective outcome reporting? (*)
10	Other	Other sources of bias	State any important concerns about bias not covered by other domains in the tool.	Was the study apparently free of other problems that could result in high risk of bias? (*)

*Items in agreement with the items in the Cochrane Risk of Bias tool.
